# Prevalence, Molecular Identification, and Risk Factors for *Cryptosporidium* Infection in Edible Marine Fish: A Survey Across Sea Areas Surrounding France

**DOI:** 10.3389/fmicb.2019.01037

**Published:** 2019-05-15

**Authors:** Gabriela Certad, Jérôme Follet, Nausicaa Gantois, Ourida Hammouma-Ghelboun, Karine Guyot, Sadia Benamrouz-Vanneste, Emilie Fréalle, Yuwalee Seesao, Baptiste Delaire, Colette Creusy, Gaël Even, Véronique Verrez-Bagnis, Una Ryan, Mélanie Gay, Cécile Aliouat-Denis, Eric Viscogliosi

**Affiliations:** ^1^CNRS, Inserm, CHU Lille, U1019 – UMR 8204 – CIIL – Centre d’Infection et d’Immunité de Lille, Institut Pasteur de Lille, Université de Lille, Lille, France; ^2^Délégation à la Recherche Clinique et à l’innovation, Groupement des Hôpitaux de l’Institut Catholique de Lille, Lille, France; ^3^ISA-YNCREA Hauts-de-France, Lille, France; ^4^CNRS, ISEN, UMR 8520 – IEMN, Université de Lille, Lille, France; ^5^Laboratoire Ecologie et Biodiversité, Faculté de Gestion Economie et Sciences, Institut Catholique de Lille, Lille, France; ^6^Service d’Anatomie et de Cytologie Pathologiques, Groupement des Hôpitaux de l’Institut Catholique de Lille, Lille, France; ^7^Gènes Diffusion, Douai, France; ^8^PEGASE-Biosciences, Institut Pasteur de Lille, Lille, France; ^9^Ifremer, Laboratoire Ecosystèmes Microbiens et Molécules Marines pour les Biotechnologies, Nantes, France; ^10^Centre for Sustainable Aquatic Ecosystems, College of Science, Health, Engineering and Education, Murdoch University, Perth, WA, Australia; ^11^Laboratory for Food Safety, French Agency for Food, Environmental and Occupational Health and Safety (ANSES), Boulogne-sur-mer, France

**Keywords:** *Cryptosporidium*, edible marine fish, 18S rRNA gene, gp60, molecular epidemiology, phylogeny, novel genotypes, European seas

## Abstract

*Cryptosporidium*, a zoonotic pathogen, is able to infect a wide range of hosts including wild and domestic animals, and humans. Although it is well known that some parasites are both fish pathogens and recognized agents of zoonosis with a public health impact, little information is available concerning the prevalence of *Cryptosporidium* in wild aquatic environments. To evaluate the prevalence of *Cryptosporidium* spp. in commercially important edible marine fish in different European seas (English channel, North sea, Bay of Biscay, Celtic sea and Mediterranean sea), 1,853 specimens were collected as part of two surveys. Nested PCR followed by sequence analysis at the 18S rRNA gene locus was used to identify *Cryptosporidium* spp. The overall prevalence of *Cryptosporidium* spp. in sampled fish reached 2.3% (35 out of 1,508) in a first campaign and 3.2% (11 out of 345) in a second campaign. Sequence and phylogenetic analysis of positive samples identified *Cryptosporidium parvum* (*n* = 10) and seven genotypes which exhibited between 7.3 and 10.1% genetic distance from *C. molnari*, with the exception of one genotype which exhibited only 0.5–0.7% genetic distance from *C. molnari.* Among 31 analyzed fish species, 11 (35.5%) were identified as potential hosts for *Cryptosporidium.* A higher prevalence of *Cryptosporidium* spp. was observed in larger fish, in fish collected during the spring-summer period, and in those caught in the North East Atlantic. *Pollachius virens* (saithe) was the most frequently *Cryptosporidium* positive species. In fish infected by other parasites, the risk of being *Cryptosporidium* positive increased 10-fold (OR: 9.95, CI: 2.32–40.01.04, *P* = 0.0002). Four *gp60* subtypes were detected among the *C. parvum* positive samples: IIaA13G1R1, IIaA15G2R1, IIaA17G2R1, and IIaA18G3R1. These *C. parvum* subtypes have been previously detected in terrestrial mammals and may constitute an additional source of infection for other animals and in particular for humans. Microscopical examination of histological sections confirmed the presence of round bodies suggestive of the development of *C. parvum* within digestive glands. We report herein the first epidemiological and molecular data concerning the detection of *Cryptosporidium* in edible marine fish in European seas surrounding France broadening its host range and uncovering potential novel infection routes.

## Introduction

The world fish production increased to 171 million tons in 2016 (compared for instance to a production of 156 million tons in 2012) with European fisheries and aquaculture representing overall 6.4 million tons. Interestingly, within the European Union, around 80% of fish consumed is wild caught with an annual fish consumption of 18.1 kg per capita (FAO, 2018).

Considering that protozoan and metazoan parasites can infest edible fish worldwide, the problem of human health risks due to wild fish ingestion is an important issue. Some of these parasites are both fish pathogens and recognized zoonotic pathogens with public health impacts but uncertainty exists about the zoonotic potential of some fish pathogens. The most important fish species in the fish industries from many countries can be infected with parasites. Furthermore, some fish parasites can affect the appearance, touch, odor, texture, temperature and taste (organoleptic properties) of fish products, negatively impacting the fish industry economy (Agence Française de Sécurité Sanitaire des Aliments [ANSES], 2010).

Even in developed countries, where sanitary infrastructures are usually good, food- or water-borne parasitic infections are frequent (Omarova et al., 2018). Indeed, the persistence of water- or foodborne outbreaks and the occurrence of infections due to emerging or re-emerging pathogens are favored by different factors related to changes in consumer life styles such as: increased consumption of fresh products, exotic food or raw or lightly cooked meat and increasing global demand for protein of animal origin, and home-meal replacement (Collins, 1997; Broglia and Kapel, 2011).

*Cryptosporidium* is a waterborne and foodborne protozoan parasite responsible for more than 8 million cases of foodborne illness annually (Ryan et al., 2018). The parasite is a cause of severe diarrhea mainly in immunocompromised people and young children but also in a wide range of vertebrates including fish, amphibians, reptiles, birds, and mammals (Ryan et al., 2014). To date, it has been genetically characterized in more than 25 species of both marine and freshwater fish. Three species of *Cryptosporidium* are recognized in fish: *Cryptosporidium molnari* (Alvarez-Pellitero and Sitjà-Bobadilla, 2002; Palenzuela et al., 2010), *C. scophthalmi* (Alvarez-Pellitero et al., 2004) and *C. huwi* (previously known as piscine genotype I) (Ryan et al., 2015). Additionally, other *Cryptosporidium* species identified in other groups of vertebrates such as *C. parvum, C. hominis, C. scrofarum* and *C. xiaoi*, have also been detected in fish. Furthermore, fifteen *Cryptosporidium* fish genotypes, and one *Cryptosporidium* rat III-like genotype, have been reported (Ryan et al., 2014; Yang et al., 2015, 2016; Couso-Pérez et al., 2018).

In fish hosts, *Cryptosporidium* is found either in the stomach or intestine. Additionally, it has been described that the parasite can cause pathological effects in fish (Alvarez-Pellitero and Sitjà-Bobadilla, 2002; Alvarez-Pellitero et al., 2004) as well as an increase in the mortality rate, mainly in juvenile fish (Murphy et al., 2009).

The majority of studies on piscine *Cryptosporidium* have been carried out on aquarium or farmed fish but scarce data is available concerning the molecular identification of *Cryptosporidium* genotypes and species in wild marine fish and, in particular, in edible fish. Actually, only two studies have been carried on in Australia and Papua New Guinea on wild marine fish (Reid et al., 2010; Koinari et al., 2013). To our knowledge, no updated information on *Cryptosporidium* infection in marine fish is available in France. In a previous study from our group, the overall prevalence of *Cryptosporidium* spp. in freshwater fish sampled from Lake Geneva (France) reached 37% (Certad et al., 2015).

Thus, the main goal of our study was to evaluate the prevalence of *Cryptosporidium* species and genotypes in selected edible fish species sampled in defined marine geographic areas surrounding France (European fishing waters). Considering that many factors related to the host and to the environment can influence host-parasites relationships (Sitjà-Bobadilla et al., 2005), we also aimed to determine the influence of host and environmental factors on *Cryptosporidium* prevalence in fish.

## Materials and Methods

### Fish Sampling

Epidemiological studies were conducted during two surveys: For the first survey, commercially important fish species were collected between 2011 and 2014 through research cruises belonging to the French Ifremer (Institut Français de Recherche pour l’Exploitation de la Mer) in different European seas as follows: the English channel, the North sea, the Bay of Biscay, the Celtic sea and the Mediterranean sea, or through purchases from wholesalers or retailers for commercial catches, and for farmed fish in the case of salmon and sea bass. Fish retailers were chosen by their representativeness of fishing areas in order to complement marine fish species according to range and origin. For the second survey, fish sampling was performed at Boulogne-Sur-Mer, the first France’s fishermen’s port, between 2014 and 2015 targeting the four most frequently caught fish species at this port: saithe, mackerel, herring, and whiting. Sampling surveys allowed catching fresh ungutted fish.

Available data about fishing area, fishing vessel, fishing date and kind of storage were recorded. Fish specimens of the selected species were sampled at various times and areas on the basis of fishery season. Fishes were analyzed immediately after been caught in the case of specimens collected through research cruises or after fish landing when obtained from retailers. When fishes were obtained from retailers, it was not always possible to pinpoint the exact location of the fishing area within the North East Atlantic. The weight, size, sex, and sexual maturity were determined for each fish. The presence of ecto and endoparasites other than *Cryptosporidium* was detected by visual examination of the body of the fish before and after evisceration. In order to classify fish according to weight and size, five groups were defined ranging from 1-smallest fishes to 5-largest fishes (Supplementary Figure S1) using a hierarchical cluster analysis with the R stats package. Fish were dissected, scrapings of the gastrointestinal epithelia were performed for each animal, the cells were preserved in RCL2^^®^^ (Alphelys, Plaisir, France) and stored at -20°C as previously described (Certad et al., 2015). Sections of the stomach and bowel were fixed in 10% buffered formalin for histological analysis. Ifremer research cruises are carried out with the French Oceanographic Fleet and are under the supervision of the French Ministry of Education and Research. A Steering Committee evaluates and approves the entire scientific campaign program before implementation. The study was performed in accordance with the EU directive 2010/63/EU and followed all the guidelines of the deontology charter of Ifremer’s research.

### DNA Extraction

Genomic DNA extraction was performed from scrapings of the gastric and intestinal epithelia on 96-well plates, using the NucleoSpin^TM^ Kit (Macherey-Nagel, GmbH & Co KG, Germany) according to the manufacturer as previously described (Certad et al., 2015). DNA was eluted in 100 μl of elution buffer.

### Nested PCR

Fish samples were tested for identification of *Cryptosporidium* at the 18S rRNA gene locus as previously described (Certad et al., 2015). Nested PCR was performed using a MJ Research PTC-200 Thermal Cycler (Marshall Scientific, Waltham, MA, United States). Secondary PCR products were visualized on a 2% agarose gel stained with Ethidium Bromide fluoresce under ultraviolet light. Although nested PCR may not accurately represent the genetic diversity originally present in the sample, this technique is often appropriate to obtain sufficient DNA copies, considering that environmental samples, and in particular gastric or intestinal tissues from fishes or feces from wildlife, frequently contain low amounts of oocysts and high levels of PCR inhibitors (Paparini et al., 2017).

### DNA Sequencing and Analysis

To identify *Cryptosporidium* species or genotypes, positive secondary nested PCR products were purified using the NucleoFast^^®^^ 96 PCR kit (Macherey Nagel, GmbH & Co KG, Germany). Purified PCR products were sequenced directly in both directions, using the secondary PCR primers (Genoscreen, Pasteur Institute of Lille, France). Obtained nucleotide sequences were aligned using the BioEdit v7.0.1 package, and compared with available DNA sequences of *Cryptosporidium* in GenBank data base using the NCBI BLAST basic local alignment search tool^1^. Subtyping of *C. parvum* was based on sequence analysis of the 60 kDa glycoprotein (*gp60*) gene as previously reported (Gatei et al., 2007). The amplified DNA fragments were purified, sequenced, and analyzed as described above. All of the nucleotide sequences identified in this study were deposited in GenBank under the accession numbers MK236538-MK236548.

### Phylogenetic Analysis

The 18S rRNA nucleotide sequences from the present study were aligned with *Cryptosporidium* sequences retrieved from GenBank (Benson et al., 2004). Sequences were aligned with MUSCLE (Elwakil and Hewedi, 2010) and the most suitable nucleotide substitutions model was selected using jModelTest2 (Darriba et al., 2012). Maximum Likelihood (ML) and Distance trees were constructed using MEGA version 7 using the Kimura 2-parameter model and gaps/missing data treatment set to complete deletion (Kumar et al., 2016). Bootstrap support was based on 1000 replications. Bayesian phylogenetic reconstructions were produced from alignments using MrBayes (Ronquist et al., 2012), with the HK85 substitution model, a MCMC length of 1,100,000, burn-in of 10,000, and subsampling every 200 iterations.

### Histological Analysis

Paraffin-embedded tissues were cut to a thickness of 5 μm and stained with hematoxylin and eosin (H & E). A Leica DMRB microscope equipped with a Leica digital camera connected to an Imaging Research MCID analysis system (MCID Software, Cambridge, United Kingdom) was used for observation of the histological sections.

### Statistical Analysis

Fisher’s exact test was used to analyze the relationship between different categorical variables. A logistic regression model was used to calculate odds ratios (OR) with *Cryptosporidium* presence as the main outcome. Multiple correspondence analysis (MCA), a data reduction technique similar to factor or principal component analysis, was applied to identify potential risk factors for *Cryptosporidium* infection in fishes. This descriptive statistical technique is valuable to analyze data and confirm associations or similarities between quantitative or qualitative variables; then, these variables are categorized without a probabilistic distribution defined *a priori* (Greenacre and Blasius, 2006). MCA facilitates the examination of different variables simultaneously; the results are represented by a graphic, and a point represents each category of every variable, and the distance from one point to another and from the center represents the relationship among the categorical variables. Risk factor variables included in the analysis were: localization of the fishing area, campaigns, seasonality, taxonomical position (order) of fish, size and weight grouping. The general significance level was set at a *P*-value below 0.05. All analyses were performed using Vassarstats software and packages stats from the R statistical computing program.

## Results

In total, 1,508 fishes were collected in the first survey: 765 onboard Ifremer research vessels and 743 from fish retailers, representing 31 different fish species. The molecular analysis of fish digestive tissues allowed the identification of *Cryptosporidium* spp. in 35 out of 1,508 fish, representing a prevalence of 2.3% (Table 1). Table 1 shows the distribution of positive *Cryptosporidium* samples according to different surveys. The fish sex had no statistically significant effect on the presence of the parasite (*P* = 0.88). Concerning the distribution of *Cryptosporidium* according to fish areas, 28 (81%) positive cases were found in specimens sampled inland from wholesalers or retailers compared to 7 (19%) in specimens sampled during research campaigns. This difference in distribution was statistically significant, with the risk of *Cryptosporidium* detection four times higher in the first group (OR: 4, CI: 1.64–8.69, *P* = 0.0001) (Table 2). No specimens from aquaculture were found positive for the parasite. Concerning seasonality, 29 (83%) *Cryptosporidium* positive cases were found in fish collected during the spring-summer period, compared to 6 (17%) during the fall-winter period, with the risk of *Cryptosporidium* presence 4-fold higher in the first group. This difference in distribution was statistically significant (OR: 4.14, CI: 1.71–10.04, *P* = 0.0007) (Table 2). Overall, 952 (63%) fishes out of 1,508 were infected by different parasites. The most frequently detected parasites were nematodes of the Anisakidae family in 56% of fishes. Other parasites were also found such as trematodes, cestodes, microsporidia, and copepods. In fish infected with other parasites, the risk of simultaneous presence of *Cryptosporidium* increased 10-fold (OR: 9.95, CI: 2.32–40.01.04, *P* = 0.0002) (Table 2).

**Table 1:** Description of campaigns for fish sampling: geographic location, seasonality, taxonomic position of fish species, and prevalence of *Cryptosporidium* (First survey).

Campaigns for fish sampling	Geographic location	Fishery season	Taxonomic position of fish species (order)	Number of sampled fish specimens	*Cryptosporidium-*positive fish number (%)
PELGAS 1	Bay of Biscay	Spring	Gadiformes	90	0 (0)
			Perciformes		
			Clupeiformes		
			Lophiiformes		
			Others^∗∗^		
PELMED1	Mediterranean sea	Summer	Gadiformes	112	4 (3.6)
			Perciformes		
			Clupeiformes		
EVHOE 1	Bay of Biscay, Celtic sea	Fall	Gadiformes	119	2 (1.7)
			Perciforme		
			Lophiiformes		
			Pleuronectiformes		
IBTS 1	English channel and North sea	Winter	Gadiformes	147	1 (0.7)
			Perciformes		
			Clupeiformes		
			Lophiiformes		
			Pleuronectiformes		
PELGAS 2	Bay of Biscay	Spring	Gadiformes	71	0 (0)
			Perciformes		
			Clupeiformes		
			Lophiiformes		
			Others^∗∗^		
PELMED 2	Mediterranean sea	Summer	Gadiformes	81	0 (0)
			Perciformes		
			Clupeiformes		
EVHOE 2	Bay of Biscay, Celtic sea	Fall	Gadiformes	145	0 (0)
			Perciformes		
			Lophiiformes		
			Pleuronectiformes		
Retailers	North East Atlantic ^∗^, Mediterranean sea, Black sea	Spring	Gadiformes	265	4 (1.5)
			Perciformes		
			Pleuronectiformes		
			Salmoniformes		
		Summer	Gadiformes	203	21 (10.34)
			Perciformes		
			Pleuronectiformes		
		Fall	Gadiformes	195	3 (1.5)
			Perciformes		
			Pleuronectiformes		
		Winter	Gadiformes	80	0 (0)
			Pleuronectiformes		
TOTAL				1508	35 (2.3)


*^∗^When fishes were collected from retailers, it was not always possible to pinpoint in detail the exact location of the fishing area within the North East Atlantic. PELGAS (PELagiques GAScone) 1, campaign 2011; PELGAS 2, campaign 2012; PELMED (PELagiques MEDiterranée) 1, campaign 2011; PELMED 2, campaign 2012; EVHOE (EValuation Halieutique de l’Ouest de l’Europe) 1, campaign 2011; EVHOE 2, campaign 2012; IBTS (International Bottom Trawl Survey), campaign 2012. ^∗∗^Beloniformes, Scorpaeniformes, Carcharhiniformes, and Tetraodontiformes.*

**Table 2:** Prevalence of *Cryptosporidium* in fishes sampled during the national survey according to host and environmental factors.

Groups of fish	Presence of *Cryptosporidium* N (%)	Absence of *Cryptosporidium* N (%)	OR	95% CI	*P*
Male^∗^	11/514 (2.1)	503/514 (97.9)	1.05	(0.50–2.2)	0.89
Female^∗∗^	19/863 (2.2)	844/863 (97.8)			
From North East Atlantic	28/713 (3.9)	685/713 (96.1)	4.6	(1.96–10.1)	<**0**.**0001**
From all other zones	7/795 (0.9)	788/795 (99.1)			
Sampling from retailers	28/743 (3.8)	714/743 (96.1)	3.77	(1.64–8.7)	**0**.**0001**
Sampling in research campaigns	7/765 (0.9)	758/765 (99.1)			
Fish order Gadiformes	29/700 (4.1)	671/700 (95.9)	5.78	(2.33–13.4)	<**0**.**0001**
All other orders	6/808 (0.7)	802/808 (99.3)			
Species *Pollachius virens*	15/80 (18.7)	65/80 (81.2)	16.24	(7.95–33.1)	<**0**.**0001**
All others species	20/1428 (1.4)	1408/1428 (98.6)			
Caught in fall-winter	6/686 (0.9)	680/686 (99.1)	4.14	(1.71–10.0)	**0**.**0007**
Caught in spring-summer	29/822 (3.5)	793/822 (96.5)			
Infected with other parasites^∗∗∗^	33/952 (3.5)	919/952 (96.5)	9.95	(2.32–40.0)	<**0**.**0001**
No detected parasites	2/556 (0.4)	554/556 (99.6)			


*^∗^, ^∗∗^The sex could not be determined for all fishes. ^∗∗∗^952 (63%) fishes out of 1,508 were infected by different parasites. The most frequently detected parasites were nematodes of the Anisakidae family in 56% of fishes. Other parasites were also found such as trematodes, cestodes, microsporidia and copepods. *P* values in bold are statistically significant.*

Overall, out of 31 analyzed fish species, 11 (35.5%) were recognized as potential new hosts for *Cryptosporidium* spp. with *Pollachius virens, Scomber japonicus* and *Molva dypterygia* the species with the highest prevalence (Table 3). In particular, the prevalence in *Pollachius virens* reached 19% and the risk of detection of *Cryptosporidium* in this host, increased 16-fold when compared with the other fish species (OR: 16.24, CI: 7.95–33.18, *P* < 0.0001) (Table 2).

**Table 3:** *Cryptosporidium* distribution in wild marine fishes identified at the 18S rRNA gene locus (national survey).

Common name	Scientific name	Fishing area	Mean fish size in cm (SD)	Fish minimum landing size (cm)^∗∗^	Mean fish weight in g (SD)	Number of *Cryptosporidium* positive individuals (%)	Number of individuals harboring identified *Cryptosporidium* species		
							
							*Cr sp*	*Cr p*	*Cr ml*
Saithe	*Pollachius virens*	North East Atlantic^∗^	51.7 ± 9.9	35	1302 ± 835	15/80 (18.8)	15	0	0
Cod	*Gadus morhua*	North East Atlantic^∗^	43.9 ± 10.3	35	1108 ± 1015	2/132 (1.5)	0	1	1
Whiting	*Merlangius merlangus*	North East Atlantic^∗^	32.7 ± 3.7	27	287.7 ± 86.13	1/128 (0.8)	1	0	0
Blue ling	*Molva dypterygia*	North East Atlantic^∗^	98.42 ± 9.8	70	3899,4 ± 853.5	3/69 (4.4)	2	1	0
Mackerel	*Scomber scombrus*	North East Atlantic^∗^	30.6 ± 6.1	30	230.6 ± 125.7 for 88 out of 91	1/91 (1.1)	0	1	0
Common sardine	*Sardina pilchardus*	Mediterranean sea	16.7 ± 4.2	11	40 ± 32.9 for 70 out of 78	1/78 (1.3)	0	1	0
Spanish Mackerel	*Scomber japonicus*	Mediterranean sea	22.5 ± 2.2	None	129.2 ± 12.9	2/31 (6.5)	0	2	0
Anchovy	*Engraulis encrasicolus*	Mediterranean sea	13.1 ± 2.1	12	15.3 ± 9.5 for 135 out of 146	1/146 (0.7)	0	1	0
Hake	*Merluccius merluccius*	North East Atlantic^∗^	38.5 ± 9.9	27	441.5 ± 452.6 for 139 out of 146	1/146 (0.7)	1	0	0
Herring	*Clupea harengus*	North East Atlantic^∗^	24.9 ± 5.0	20	151,8 ± 94.6	1/106 (0.9)	0	1	0
Ling	*Molva molva*	North East Atlantic^∗^	78.4 ± 20.0	63	3309.5 ± 3076	7/46 (1)	7	0	0
Angler	*Lophius piscatorius*	Bay of Biscay, English Channel, North sea	40.8 ± 14.2	None	1323 ± 4723.8	0/53 (0)	0	0	0
European seabass	*Dicentrarchus labrax*	Bay of Biscay, English Channel, North sea Aquaculture (Mediterranean, Black sea and Norwegian sea)	36.2 ± 4.5	42	535.5 ± 288.3	0/106 ^∗∗∗^ (0)	0	0	0
Haddock	*Melanogrammu aeglefinus*	North East Atlantic^∗^, Bay of Biscay, English Channel, North sea	28.2 ± 11.0	30	345 ± 337.2	0/90 (0)	0	0	0
European plaice	*Pleuronectes platessa*	North East Atlantic^∗^, English Channel, North sea	35.7 ± 6.03	27	534.9 ± 302.4	0/32 (0)	0	0	0
Salmon	*Salmo salar*	Aquaculture (North East Atlantic^∗^)	35.7 ± 6.0	None	2330 ± 517.6	0/40 (0)	0	0	0
Common sole	*Solea solea*	North East Atlantic^∗^, English Channel, North sea	28.6 ± 3.8	24	240.5 ± 114.0	0/106 (0)	0	0	0
Garfish	*Belone belone*	Bay of Biscay	83	35	862.5	0/2 (0)	0	0	0
Spotted seabass	*Dicentrarchus punctatus*	Bay of Biscay	31	None	250	0/1 (0)	0	0	0
Gurnard	*Eutrigla gurnardus*	Bay of Biscay	21	None	60	0/1 (0)	0	0	0
School shark	*Galeorhinus galeus*	Bay of Biscay	76	None	76	0/1 (0)	0	0	0
Megrim	*Lepidorhombus whiffiagonis*	Bay of Biscay	39	20	485	0/1 (0)	0	0	0
Common dab	*Limanda limanda*	English Channel, North sea	26	None	95	0/1 (0)	0	0	0
Blackbellied angler	*Lophius budegassa*	Bay of Biscay	39.3 ± 11.2	None	482 ± 156.1	0/4 (0)	0	0	0
Blue whiting	*Micromesistius poutassou*	Bay of Biscay	30	None	165	0/1 (0)	0	0	0
Ocean sunfish	*Mola mola*	Bay of Biscay	41	None	4920	0/2 (0)	0	0	0
European Pollock	*Pollachius pollachius*	English Channel, North sea	66	None	3604	0/3 (0)	0	0	0
Atlantic bonito	*Sarda sarda*	Bay of Biscay	37	None	537.5	0/3 (0)	0	0	0
Black seabream	*Spondyliosoma cantharus*	Bay of Biscay	28.5	None	435	0/2 (0)	0	0	0
Mediterranean horse mackerel	*Trachurus mediterraneus*	Mediterranean sea	23	None	85	0/1 (0)	0	0	0
Pouting	*Trisopterus luscus*	Bay of Biscay	29.6 ± 2.9	None	315.5 ± 78.1	0/5 (0)	0	0	0
Total of positive fishes						35/1508 (2.3)	26	8	1


*^∗^When fishes were collected from retailers, it was not always possible to pinpoint in detail the location of the fishing area within the North East Atlantic. ^∗∗^The obligation to land all fishes only applies to species for which there is a quota or a total allowable catch. ^∗∗∗^Aquaculture: *N* = 30. Cr sp, *Cryptosporidium* sp; Cr p, *C. parvum;* Cr ml, *C. molnari-like* genotype.*

A higher prevalence of *Cryptosporidium* was recorded in larger fishes according to weight and size grouping, with a tendency to decrease in smaller fishes.

**FIGURE 1:**
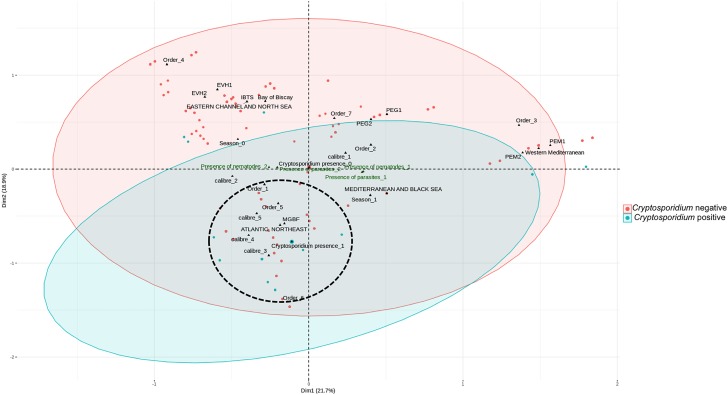
Multiple correspondence analysis. The cluster analysis explained 41% of the total variation. Variables which are closest to each other and distant from the center on the scatterplot are the most likely related. Even if these variables are not grouped as perfect clusters, most *Cryptosporidium* positive fishes are in the zone encircled (dot lines) that we called cluster 1. In general, this cluster contained larger fishes mainly of size and weight groupings (calibers) 4 and 5, from the order Gadiformes, caught in the North East Atlantic. PELGAS (PELagiques GAScone) 1, campaign 2011; PELGAS 2, campaign 2012; PELMED (PELagiques MEDiterranée) 1, campaign 2011; PELMED 2, campaign 2012; EVHOE (EValuation Halieutique de l’Ouest de l’Europe) 1, campaign 2011; EVHOE 2, campaign 2012; IBTS (International Bottom Trawl Survey), campaign 2012; MGBF, Retailers. Season 0, fall/winter; Season 1, spring/summer. Order 1, Gadiformes; Order 2, Perciformes; Order 3, Clupeiformes; Order 4, Lophiiformes; Order 5, Pleuronectiformes; Order 6, Salmoniformes; Order 7, others. Fishes were classified according to weight and size and five groups were defined ranging from 1-smallest fishes to 5-largest fishes (Supplementary Figure S1) using a hierarchical cluster analysis with the R stats package.

The MCA (Figure 1) shows the coordinates of each variable on the two dimensions which explains the largest percentage of the variance in the data. Variables which are closest to each other on the scatterplot and far from the center of the plot are the most closely related. Even if these variables are not grouped as perfect clusters, most *Cryptosporidium* positive fishes are in the zone called cluster 1. In general, this cluster contained larger fishes mainly of size and weight groupings 4 and 5, from the Gadiformes order, caught in the North East Atlantic.

**FIGURE 2:**
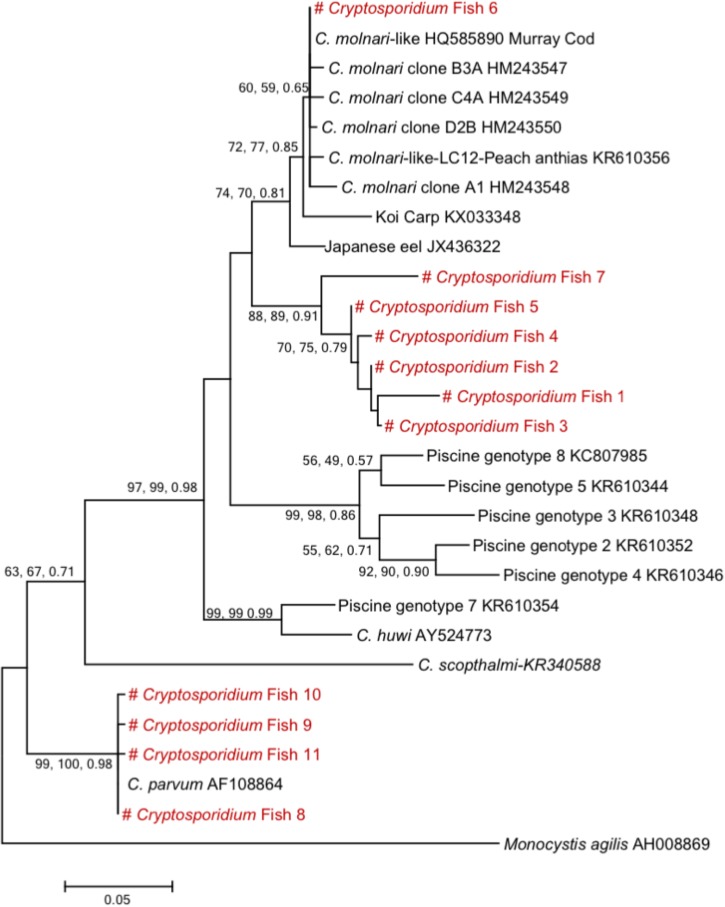
Phylogenetic tree showing the evolutional relationships of *Cryptosporidium* piscine isolates inferred by ML analysis of 18S rRNA gene sequences. Percentage support (>50%) from 1,000 pseudoreplicates from ML and distance analyses and posterior probabilities from Bayesian analysis are indicated at the left of the supported node. Red texts correspond to the sequences from this study.

Sequence and phylogenetic analysis (Figure 2) at the 18S rRNA gene locus identified one species of *Cryptosporidium* and seven genotypes, distributed as follows: 8 (22%) *C. parvum*, 16 (45.7%) belonged to a novel genotype (#Cryptofish1) that exhibited 7.3–8.5% genetic distance from *C. molnari* (HM243547- HM243550), 6 (17.1%) belonged to another genotype (#Cryptofish 2) that exhibited 8.5–9.5% genetic distance from *C. molnari*, 2 (5.7%) belonged to a new genotype (#Cryptofish 3) which exhibited 8.9–10.1% genetic distance from *C. molnari*, single isolates (#Cryptofish 4 and # Cryptofish 5) (5.7%) which exhibited 8.2–9.5% and 7.6–8.8% genetic distance from *C. molnari*, respectively, and one (2.9%) (#Cryptofish 6) which exhibited 0–0.5% genetic distance from *C. molnari*-like isolates (HQ585890 and KR610356) and 0.5–0.7% genetic distance from *C. molnari*. Estimates of evolutionary divergence between sequences are shown in Supplementary Figure S2.

**FIGURE 3:**
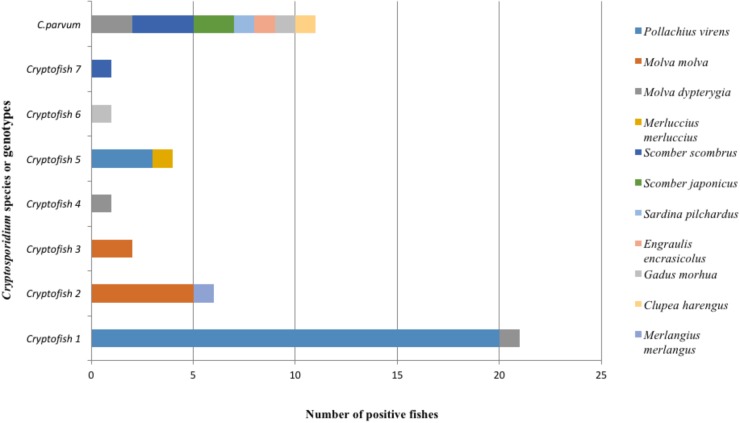
Global distribution of different types of *Cryptosporidium* sequences identified at the 18S rRNA gene locus according to fish species found as hosts in both surveys (*n* = 46). Eleven new species of fish were identified as potential hosts for *Cryptosporidium.* Cryptofish 1 was the most frequently identified novel genotype. *Cryptosporidium* genotypes had less host diversity when compared to *C. parvum* which was found in seven different fish species.

**FIGURE 4:**
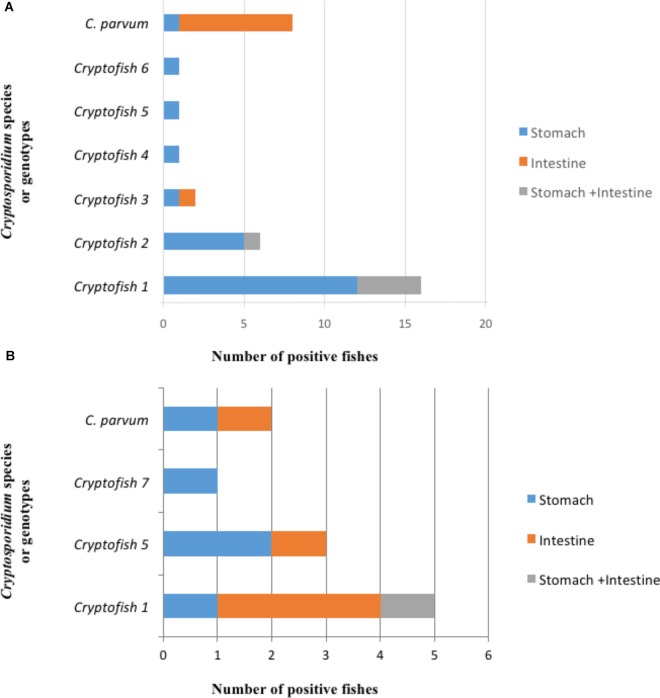
Distribution of different types of *Cryptosporidium* sequences identified at the 18S rRNA gene locus according to the anatomical location. **(A)** First survey (*n* = 35). *C. parvum* was identified in the intestine of 7 fishes and in the stomach of one fish, while the *C. molnari-*like genotype (#Cryptofish 6) was found in the stomach of one fish. The various novel *Cryptosporidium* genotypes were detected in the stomach of 20 fish, in the intestine of one fish and in both the intestine and stomach of 5 fish. **(B)** Second survey (*n* = 11). *C. parvum* was identified in the bowel of one fish and the stomach of one fish. The 3 novel *Cryptosporidium* genotypes were identified simultaneously in the stomach and bowel of one fish, in the stomach only of four fishes, and in the intestine only of four fishes.

The distribution of *Cryptosporidium* species and genotypes according to the species of fish identified as hosts is shown in Figure 3. Molecular analysis of DNA extracted from each fish stomach or intestine, followed by nested 18S rRNA PCR and sequencing allowed the identification of *C. parvum* in the intestine of 7 fish and in the stomach of one fish, while the *C. molnari*-like genotype (#Cryptofish 6) was found in the stomach of one fish. The various novel *Cryptosporidium* genotypes were detected in the stomach of 20 (76.9%) fish, in the intestine of one (3.8%) fish and in both the intestine and stomach of 5 (19.2%) fish. For the latter case, the same genotypes were found in both organs for each fish. In Figure 4A, the distribution of species/genotypes according to anatomical location is shown. Four *gp60* subtypes were identified among the *C. parvum* positive samples: IIaA13G1R1, IIaA15G2R1, IIaA17G2R1, and IIaA18G3R1.

**FIGURE 5:**
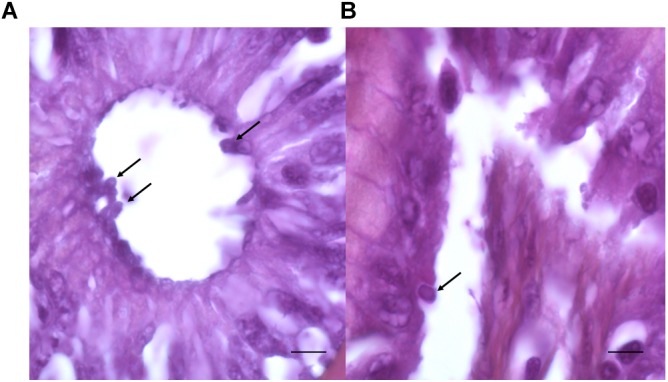
Stained sections of the intestinal tract of fishes. **(A**,**B)** Presence of round bodies suggestive of the developmental stages of *C*. *parvum* observed in the apical position (arrows) within the intestinal epithelial cells. Bars = 15 μm **(A)** and 5 μm **(B)**.

After examination of histological sections from the digestive tract, the presence of *Cryptosporidium*-like bodies within the cells of the intestinal epithelium and in apical position was detected in one *C. parvum*-positive fish (Figure 5). The presence of parasites could not be studied in all positive fishes due to considerable lysis of tissues.

Concerning the second survey, in total, 345 fishes were collected in this regional survey, from fishermen at Boulogne-Sur-Mer. *Cryptosporidium* exhibited a prevalence of 3.2% (11/345) in edible fish. The parasite was only detected in *Pollachius virens* and *Scomber scombrus*, with *Pollachius virens* the species with the highest prevalence (Table 4), confirming data collected from the national survey. After genotyping, *C. parvum* was identified in two fishes. Additionally, genotype # Cryptofish 1 was identified in six isolates, genotype # Cryptofish 5 in three and another novel genotype #Cryptofish 7 which exhibited 9.1–10.4% genetic distance from *C. molnari* was identified in one isolate (Figure 2). Distribution of the different types of *Cryptosporidium* sequences identified in their corresponding host species is shown in Figure 3. Unfortunately, *C. parvum* samples from this survey were unsuccessfully subtyped by sequence analysis of the *gp60*.

**Table 4:** *Cryptosporidium* distribution in wild marine fishes identified at the 18S rRNA gene locus (regional survey).

Common name	Scientific name	Fishing area	Mean fish size in cm (SD)	Fish minimum landing size (cm)	Mean fish weight in g (SD)	Number of *Cryptosporidium* positive individuals (%)	Number of individuals harboring identified *Cryptosporidium* species		
							
							*Cr sp*	*Cr p*	*Cr ml*
Saithe	*Pollachius virens*	Eastern English channel, Norwegian sea	49.42 ± 8.8	35	114.8 ± 53.8	9/80 (11.3)	8	0	0
Mackerel	*Scomber scombrus*	Eastern English channel	30.21 ± 3.5	30	237.8 ± 99.1	2/110 (1.8)	0	3	0
Herring	*Clupea harengus*	Eastern English channel, Southern North sea	28.32 ± 3.8	20	200.2 ± 79.4	0/60 (0)	0	0	0
Whiting	*Merlangius merlangus*	Central North sea	31.35 ± 4.4	27	263.3 ± 116.2	0/95 (0)	0	0	0
Total						11/345 (3.2)	8	3	0


*Cr sp, *Cryptosporidium* sp; Cr p, *C. parvum;* Cr ml, *C. molnari-*like genotype.*

The selective extraction of DNA from the organs allowed the identification of *C. parvum* in the bowel of one fish and the stomach of one fish. The various novel *Cryptosporidium* genotypes were identified simultaneously in the stomach and bowel of one fish, in the stomach only of four fishes, and in the intestine only of four fishes. In Figure 4B, the distribution of species/genotypes according to anatomical location is shown.

## Discussion

This is the first epidemiological and molecular data on the presence of *Cryptosporidium* in edible marine fishes in European waters. Overall, the prevalence of *Cryptosporidium* spp. in sampled fish reached 2.3% in the first campaign and 3.2% in the second campaign. A lower *Cryptosporidium* prevalence in marine fish was found in Western Australia (0.8%) (Reid et al., 2010) and in Papua New Guinea 1.4% (Koinari et al., 2013).

A higher prevalence of *Cryptosporidium* was found in fishes from retailers. As explained before, fish retailers were chosen to complement marine fish species, as the fishing areas covered by them were the same as the research campaigns. One hypothesis to explain the higher prevalence in retail fishes would be that thanks to retailers the collection of all *Pollachius virens* specimens sampled for this study was possible. This fish species had the highest *Cryptosporidium* prevalence contributing to an increase in the overall prevalence of specimens sampled inland. *Pollachius virens* may be more susceptible to this parasite for unknown reasons, further studies need to be done to clarify this aspect. It is not likely that holding the fish prior to processing or the handling itself affected parasite abundance since multiple measures to avoid cross contamination between specimens were taken as a precaution in each experimental step, as described in the materials and methods section.

Previously, other studies described a high prevalence of *Cryptosporidium* spp. in marine fish, but mainly in juveniles. In cultured marine fish, the prevalence of *C. molnari* in *Dicentrarchus labrax* and *Sparus aurata* was 50 and 95% in hatcheries and 58 and 65% in the ongrowing systems, respectively (Sitjà-Bobadilla et al., 2005). Prevalence rates of up to 100% for *C. scophthalmi* were also reported in juvenile turbot (*Psetta maxima*) (Alvarez-Pellitero et al., 2004). Interestingly, in the present study *Cryptosporidium* was not detected in fishes sampled from aquaculture. The absence of parasite species in fishes from aquaculture has been attributed to the strengthened immunity of properly raised fish in aquaculture systems (Mladineo and Poljak, 2014). In addition, farmed fishes are usually fed with fish controlled food. This practice may likely decrease the risk for parasite transmission. In mammals, including calves, pigs and humans, it has been reported that *Cryptosporidium* infections are usually more frequent in neonates and young individuals and less prevalent in adults (Ryan et al., 2014). Due to our sampling method, according to size and weight (and sexual maturity when this parameter could be determined) all the analyzed fishes including those from aquaculture were adults (Tables 3, 4).

In addition, the highest prevalence of *Cryptosporidium* spp. was observed in larger fishes according to the weight and size groupings. A previous study reported a *Cryptosporidium* prevalence of 5% in adult farmed rainbow trout (*Oncorhynchus mykiss*), but a higher *Cryptosporidium* prevalence of 14% was observed in younger animals (Couso-Pérez et al., 2018). As juvenile fish were not analyzed in the present study, the *Cryptosporidium* prevalence may be underestimated. However, the higher prevalence in larger fish in the present study could be due to parasite accumulation during the lifetime of the fish, as it has been reported for other parasites such as *Anisakis* (Valero et al., 2000), and longer exposure to contaminated water (Couso-Pérez et al., 2018) or more consumption of prey potentially carrying parasites.

Interestingly, 35.5% of selected sampled species were positive for *Cryptosporidium*, and eleven new species of fish were identified as potential hosts for *Cryptosporidium* (Tables 3, 4) increasing the host range for this waterborne parasite. Among these fish hosts the most commonly *Cryptosporidium* positive were: *Pollachius virens* (saithe), *Gadus morhua* (cod) and *Molva dypterygia* (blue ling) belonging to the order Gadiformes and *Scomber scombrus* (mackerel) belonging to the order Perciformes. As already mentioned above, *Pollachius virens* was the species in which the parasite was most frequently detected in both national and regional campaigns, with a 16-fold higher risk of being *Cryptosporidium* positive. Interestingly, in these hosts, one type of sequence (#Cryptofish1) was recorded in the majority of animals either in the national or the regional campaign. The behavior of these fish species could influence parasite transmission. As gadid fishes are social and gregarious (Anders et al., 2017), parasite transmission could occur during cohabitation in dense groupings. This type of transmission was reported in a population of reared fishes experimentally infected with *C. molnari* (Sitjà-Bobadilla and Alvarez-Pellitero, 2003). Moreover, as the fish species included in our survey were predators, *Cryptosporidium* could be transmitted to other fishes through the food chain. For example, Mendez-Hermida (Méndez-Hermida et al., 2007) reported the potential role of the live microcrustacean *Artemia salina* as a *Cryptosporidium* vehicle for piscine fishes. As fishes in the present study were sampled mainly off shore, contamination through coastal water would appear to be less significant. However, in some fish species, particularly those caught in the Mediterranean sea, and in the case of intertidal fish (moving in and out of the seashore), this kind of transmission cannot be excluded.

One species of *Cryptosporidium* (*C*. *parvum)* and seven genotypes were detected in the present report. The 18S rRNA gene sequences of all *C*. *parvum* isolates identified in the present study in either the national or the regional campaign were 99–100% identical to those of *C*. *parvum* GenBank reference sequences. The genotypes, however, exhibited substantial genetic distances from *C. molnari* (7.3–10.1%), with the exception of one genotype (#Cryptofish 6) which exhibited 0.5–0.7% genetic distance from *C. molnari* and was 100% identical to *C. molnari*-like genotypes from Peach anthias (*Pseudanthias dispar*) (KR610356) and Murray Cod (HQ585890). This “*C. molnari*-like genotype” has been previously described (Zanguee et al., 2010), and more recently has also been characterized at the actin locus (Yang et al., 2015), where it exhibited 7.3–8.7% genetic distance from *C. molnari* and is therefore likely to be a novel species. Similarly, the large genetic distances of genotypes #1–5 and #7 from *C. molnari* also indicate that they are representative of novel species, even if further analysis at the actin locus is required for confirmation. The high diversity of *Cryptosporidium* species and genotypes identified in fish in the present and in other studies indicate a long-term association of *Cryptosporidium* to their fish hosts. In addition, when considering the spectrum of fish hosts, *C. parvum* seems to have a broad host range (Figure 3).

Seasonality of *Cryptosporidium* spp distribution was observed in the present study, with maximal prevalence occurring in spring and summer. Consistently, a seasonal distribution of *C. molnari* was also described in farmed gilthead seabream in Spain, with maximal parasite intensity and prevalence during the same seasons (Sitjà-Bobadilla et al., 2005). Variations in the intensity of parasitic infections linked to seasonality have been described in marine ecosystems (Wilson et al., 2001) and seasonal changes may influence the physiology of the host including the immune function or the intensity of feeding. This correlation has been also reported in natural infections of different fishes (Sitjà-Bobadilla et al., 2005) and in other *Cryptosporidium* infected non-piscine hosts including humans (Jagai et al., 2009; Wells et al., 2015), however, it is difficult to know if the seasonality found in the present study could be attributed to the infection or more likely to different fish species caught at each season.

The identification of *C*. *parvum* among edible fish hosts is of public health significance, as this species is the most common source of zoonotic infections (Ryan et al., 2014). Previously, studies in Papua New Guinea, Australia and Spain also described the detection of *C*. *parvum* in fishes (Reid et al., 2010; Koinari et al., 2013; Certad et al., 2015; Couso-Pérez et al., 2018). The presence of *C*. *parvum* in fish samples, and in particular the IIa subtype (IIaA13G1R1, IIaA15G2R1, IIaA17G2R1, and IIaA18G3R1) is maybe related to water contamination by animal and human wastes. Indeed, the zoonotic *C*. *parvum* IIa subtype family has mainly been reported in humans and calves in Europe, North America, and Australia (Xiao, 2010; Follet et al., 2011). Subtypes IIaA17G2R1 and IIaA15G2R1 were identified in North Atlantic fishes and are the same subtypes that were previously identified in the Geneva lake, France (Certad et al., 2015).

*Cryptosporidium parvum* subtypes IIaA18G3R1 and IIaA15G2R1 have been detected in foals in Brazil (Inácio et al., 2017) and are also common subtypes in both humans and cattle worldwide including Australia (Zahedi et al., 2016a). The subtype IIaA13G1R1 has been identified in lambs, goats, and wild boars in Spain (Díaz et al., 2018).

In a previous study from our group, the presence of *C*. *molnari* was detected by nested 18S rRNA PCR and sequencing in freshwater European perch (*Perca fluviatilis)* filets (Certad et al., 2015). We suggested that filet contamination with the parasite could occur after handling the fish. Although there is no evidence of transmission of *Cryptosporidium* from fish hosts to mammals (Certad et al., 2010), the presence of the parasite in filets highlighted the risk of *Cryptosporidium* infection to humans, either during the preparation process of fish or when consuming uncooked or undercooked fish carrying zoonotic species of this parasite.

The findings of the present study are important from a public health point of view considering that some of these fish species are hosts for *C. parvum* including pilchards, anchovies, and herrings which are commonly eaten after only a slight preparation (for example salted or marinated), and even without gutting (Sánchez-Monsalvez et al., 2005; Mladineo and Poljak, 2014).

In addition, it was reported that fishermen were at risk of cryptosporidiosis after fishing and consuming captured fish (Roberts et al., 2007) and that *C*. *parvum* oocysts could be transferred to persons handling blue crabs (Graczyk et al., 2007). Moreover, immunosuppressed patients are also at risk of *Cryptosporidium* infection, either by consumption of raw or undercooked fish or by contact with fish during preparation and handling (McOliver et al., 2009).

Since *C. parvum* is a zoonotic species, fish carrying this species are a potential source of infection for other animals and in particular for humans, and may also contribute to the contamination of the aquatic ecosystem. Nevertheless, it is not still clear if *C. parvum* can cause a true infection and multiply in fish hosts. The analysis of digestive histological sections from a *C. parvum*-positive marine fish however, allowed the identification of intracellular round bodies in apical position suggestive of *C. parvum* developmental stages on epithelial cells. No signal was detected when immunofluorescence using an anti-*Cryptosporidium* antibody (Crypto Cel immunofluorescence test, Cellabs, Brookvale, New South Wales, and Australia) was performed to confirm the presence of the parasite in fish tissues. This failure was probably due to the fact that formalin progressively cross-links proteins of the parasite, particularly after long time of formalin fixation (Barugahare et al., 2011). Further studies have to be done to confirm this aspect.

In conclusion, this study provides the first epidemiological data regarding the presence of *Cryptosporidium* in marine edible fish in European waters. New fish species were identified as hosts for this parasite. In addition, the influence of host factors such as the species, the weight and size groupings and environmental factors such as geographical localization or seasonality were shown to have an impact on parasite infection. Finally, the detection of zoonotic *Cryptosporidium* in fish suggests that the parasite may represent a sentinel for environmental contamination. Since wildlife can potentially contribute to *Cryptosporidium* contamination of water systems, the identification of the sources/carriers of zoonotic strains is needed for accurate risk assessment (Zahedi et al., 2016b). In addition, as the consumption of raw or thermally inadequately treated fishery products represents novel trends in human eating habits, strategic research in the field is extremely important. Further studies are required to confirm the species status of the seven novel genotypes identified.

## Author Contributions

GC, JF, KG, EF, VV-B, MG, CA-D, and EV conceived and designed the experiments. NG, OH-G, SB-V, YS, BD, and CC performed the experiments. GC, JF, NG, GE, UR, and EV analyzed the data. GE, VV-B, MG, and CA-D contributed to reagents, materials, and analysis tools. GC and UR wrote the manuscript.

## Conflict of Interest Statement

The authors declare that the research was conducted in the absence of any commercial or financial relationships that could be construed as a potential conflict of interest.

## Footnotes

1 http://www.ncbi.nlm.nih.gov/BLAST/

## Supplementary Material

FIGURE S1 Hierarchical cluster analysis was used to define variables (R stats package). Five groups were defined ranging from 1-smallest fishes to 5-largest fishes.

FIGURE S2 The number of base substitutions per site from between sequences are shown. Analyses were conducted using the Tamura 3-parameter model. The analysis involved 29 nucleotide sequences. All positions with less than 95% site coverage were eliminated. That is, fewer than 5% alignment gaps, missing data, and ambiguous bases were allowed at any position. There were a total of 371 positions in the final dataset. Evolutionary analyses were conducted in MEGA6.
