# Supplementary figures and images for: Prevalence, Molecular Identification, and Risk Factors for Cryptosporidium Infection in Edible Marine Fish: A Survey Across Sea Areas Surrounding France
Source: Front Microbiol. 2019 May 15;10:1037. doi: 10.3389/fmicb.2019.01037 (PMC6530514; doi:10.3389/fmicb.2019.01037)

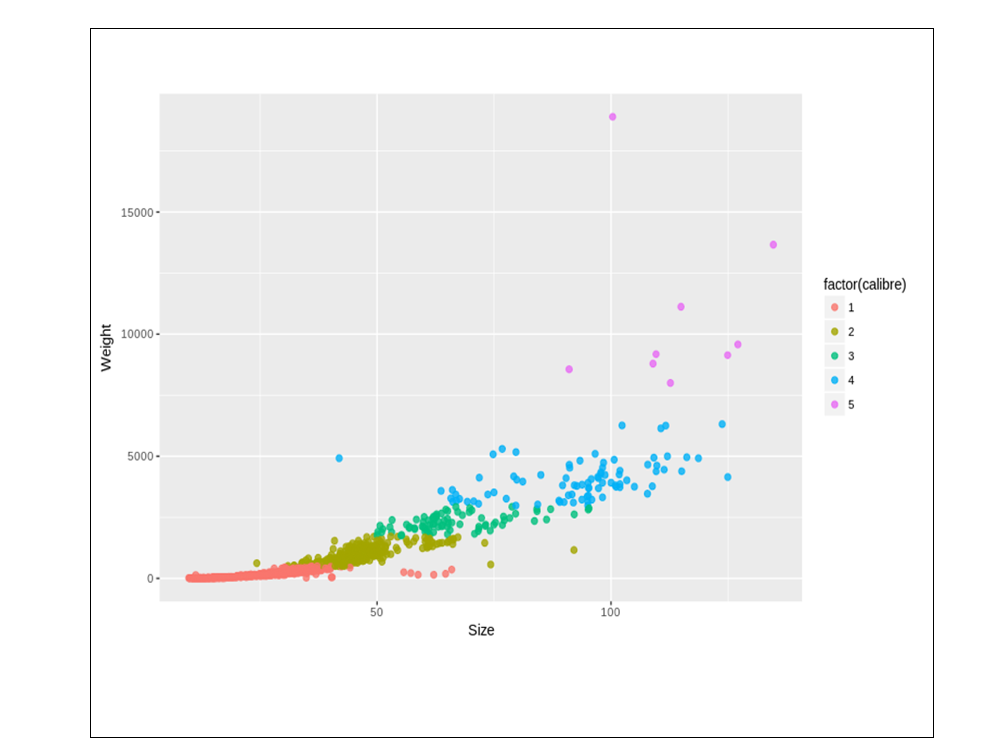

Supplement: FIGURE S1 — Hierarchical cluster analysis was used to define variables (R stats package). Five groups were defined ranging from 1-smallest fishes to 5-largest fishes. [file Image_1.TIFF]

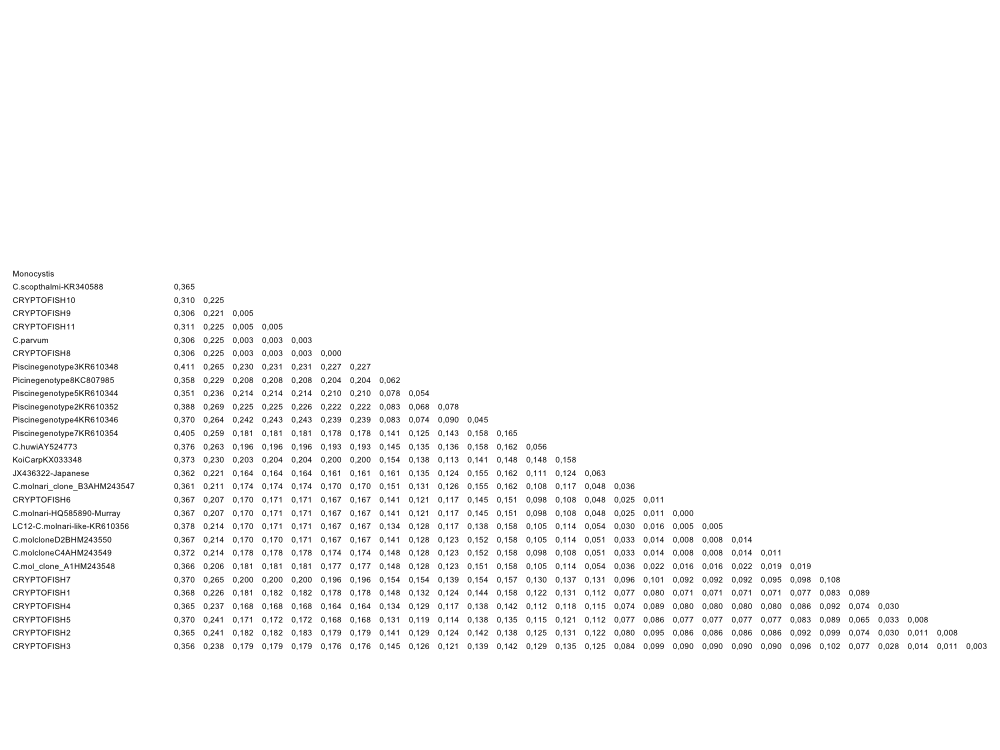

Supplement: FIGURE S2 — The number of base substitutions per site from between sequences are shown. Analyses were conducted using the Tamura 3-parameter model. The analysis involved 29 nucleotide sequences. All positions with less than 95% site coverage were eliminated. That is, fewer than 5% alignment gaps, missing data, and ambiguous bases were allowed at any position. There were a total of 371 positions in the final dataset. Evolutionary analyses were conducted in MEGA6. [file Image_2.TIFF]
